# Phosphorylation of cAMP‐Activated Exchange Protein‐1 Participates in Neuroprotection and Ferroptosis Regulation Following Intracerebral Hemorrhage in Rats

**DOI:** 10.1111/cns.70373

**Published:** 2025-04-09

**Authors:** Guannan Jiang, Jialei Zhou, Yan Zhuang, Siyuan Yang, Gang Chen, Wanchun You, Xiang Li

**Affiliations:** ^1^ Department of Neurosurgery and Brain and Nerve Research Laboratory The First Affiliated Hospital of Soochow University Suzhou China; ^2^ Institute of Stroke Research Soochow University Suzhou China; ^3^ Department of Neurosurgery The Affiliated Hospital of Jiangsu University Zhenjiang China

**Keywords:** cAMP‐activated exchange protein‐1, ferroptosis, intracerebral hemorrhage, neuroprotection, phosphorylation

## Abstract

**Background:**

Intracerebral hemorrhage (ICH) is a severe condition characterized by elevated mortality and disability rates. The cAMP‐activated exchange protein‐1 (EPAC‐1) is implicated in various cytoprotective mechanisms; however, its specific role in ICH remains unclear.

**Methods:**

A rat model of ICH was established by injecting autologous blood, while the in vitro primary neuronal model was stimulated using oxyhemoglobin (OxyHb). The construction of EPAC‐1 overexpression wild‐type (WT) and phosphorylated mutant plasmids (S108A or S108E), as well as lentiviruses, was performed for in vitro and in vivo studies.

**Results:**

The cAMP signaling pathway was found to be significantly enriched following ICH by high‐throughput sequencing analysis. Our findings showed that while EPAC‐1 protein levels remained relatively unchanged after ICH, RabGEF activity was conspicuously upregulated. This was accompanied by a marked decrease in EPAC‐1 phosphorylation levels. Mutations that activate EPAC‐1 phosphorylation led to significant improvements in neuronal survival and behavioral outcomes after ICH. Bioinformatics analysis revealed that ferroptosis was significantly enriched after ICH and showed a positive correlation with EPAC‐1 serine phosphorylation. EPAC‐1 phosphorylation activating mutations inhibit neuronal ferroptosis, whereas inactivating mutations exacerbate it.

**Conclusion:**

The phosphorylation of EPAC‐1 is essential for maintaining neuronal survival, which may be related to ferroptosis inhibition after ICH.

## Graphical abstract

The GSE264394 dataset analysis revealed cAMP signaling pathway enrichment after ICH, implicating EPAC‐1 involvement. A rat ICH model and an in vitro neuronal model were used to assess EPAC‐1 phosphorylation. Mutant plasmids and lentiviruses modulated EPAC‐1 phosphorylation, while neuronal activity, cell death, and ferroptosis were evaluated in vitro and in vivo.

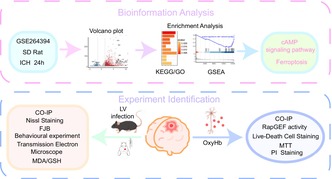

**Abbreviations**
cAMP: cyclic adenosine monophosphate
EPAC‐1: cAMP‐activated Exchange protein‐1
GEF: guanine nucleotide exchange factor
GO: gene ontology
GSH: glutathione
ICH: intracerebral hemorrhage
KEGG: Kyoto Encyclopedia of Genes and Genomes
MDA: malondialdehyde
OxyHb: oxyhaemoglobin
PKA: protein kinase A
SD: Sprague–Dawley
Ser: serine

## 1 Introduction

Intracerebral hemorrhage (ICH) arises from the bursting of brain arteries, resulting in the seepage of blood into the brain tissue and triggering harm to the neural tissue and impairment of neurological function [1]. It is associated with high morbidity, mortality, and disability rates [2, 3]. Approximately 2.2 million people worldwide experience ICH annually, accounting for 10%–20% of all stroke cases [4]. As many as 58% of individuals with ICH succumb to the condition within the first 12 months following their diagnosis, while a substantial proportion, approximately two‐thirds, of those who survive are left grappling with significant neurological impairments [5]. Despite advances in diagnosis and treatment, ICH remains a significant clinical challenge, imposing a heavy burden on society and families [6]. Therefore, understanding the complex molecular changes underlying ICH and its significant variability is crucial for developing effective interventions and improving clinical treatments [7].

The cAMP signaling pathway was found to be significantly enriched following ICH, as demonstrated by high‐throughput sequencing analysis. The cyclic adenosine monophosphate (cAMP) signaling pathway plays a critical role as an intracellular regulator, influencing a wide range of physiological processes, such as metabolism, cell proliferation, differentiation, and apoptosis [8, 9]. The cAMP‐activated exchange protein‐1 (EPAC‐1) is directly activated by cAMP and modulates numerous cellular functions [10]. Studies have shown that EPAC‐1 regulates cell adhesion and migration by activating Rap1 GTPase and promotes insulin secretion by modulating calcium channels and exocytosis, with a particularly significant role in pancreatic β‐cells [11, 12]. Additionally, EPAC‐1 influences cardiac contractile and relaxation functions by regulating calcium handling in cardiomyocytes and plays a crucial role in pathological conditions such as cardiac hypertrophy and arrhythmias [13]. EPAC‐1 is also involved in inflammatory responses, particularly in monocytes and macrophages, where it affects the inflammatory process by regulating the secretion of inflammatory cytokines and the activation of immune cells [14]. Furthermore, EPAC‐1 modulates vascular tone by affecting the relaxation and contraction of vascular smooth muscle, thereby influencing blood pressure and blood flow [15].

However, the mechanisms of EPAC‐1 in ICH remain unclear. This study explores the neuroprotective effects of EPAC‐1 phosphorylation following ICH and its underlying mechanisms. Our findings indicate that EPAC‐1 phosphorylation may act as a promising protective mechanism to mitigate secondary brain injury caused by ICH, providing a potential therapeutic strategy for its treatment. In brief, research on EPAC‐1 in ICH suggests that effective therapeutic strategies may be developed to mitigate injury after ICH by modulating EPAC‐1 phosphorylation.

## 2 Methods

### 2.1 Animals

Male Sprague–Dawley (SD) rats, with an age range of 6–8 weeks and body weight between 250 and 300 g, were kept in a specialized animal housing area, where they experienced a consistent 12‐h light and 12‐h dark schedule. Adequate supplies of water and nourishment were made available to them at all times. All experimental procedures involving animals were performed in adherence to the guidelines outlined in the National Institutes of Health's publication, *The Guide for the Care and Use of Laboratory Animals*, and received ethical clearance from the Ethics Committee of the First Affiliated Hospital of Soochow University under ethics protocol number 2018‐197.

### 2.2 ICH Model

The ICH rat model was developed following the established method [16]. The rats were induced into a state of anesthesia using 3% isoflurane via a gas anesthesia apparatus and positioned supinely within a stereotaxic apparatus (Anhui Zhenghua Biological Equipment Co. Ltd., China). Following a median scalp incision, the cranium was revealed, and perforations were created above the right‐side basal ganglia, specifically 0.2 mm posterior to the fontanelle and 3.5 mm laterally. A micro‐syringe loaded with 100 μL of the rat's own arterial blood was cautiously inserted to a depth of 5.5 mm and the blood was administered at a pace of 10 μL/min. The rats' heart rates were tracked throughout the procedure, while their body temperatures were kept at a steady 37.0°C ± 0.5°C. The Sham group received an equivalent volume of saline solution, while the remaining procedures were performed as previously described.

### 2.3 Primary Cortical Neuron Cultures

The cerebral cortex was extracted from the rat fetuses, with the protective membranes and vascular structures excised. Subsequently, the cortical segment underwent enzymatic treatment using a 0.25% solution of trypsin–EDTA for a duration of 5–8 min at 37°C. Post‐digestion, the medium was discarded, and the tissue was re‐suspended in a full culture medium. Following this, the resulting brain mixture was passed through a filter and subjected to centrifugation at a speed of 1500 r/min for 5 min. The supernatant was then re‐suspended and planted into culture dishes that had been previously prepared with a coating, after which they were placed in an incubator for a period of 5–7 days. According to previous studies, the administration of OxyHb can imitate ICH injury in vitro [17].

### 2.4 Lentiviral and Plasmids Transduction

Transfection using lateral ventricular injection of lentiviral [18]. Rats were anesthetized and secured on a stereotaxic apparatus 10 days before ICH surgery. A hole was created 1.5 mm away from the fontanelle laterally and 1.1 mm caudally, followed by a gradual administration of the lentiviral solution into the lateral ventricle at a depth of 3.5 mm, with an injection pace of 0.5 μL/min. Three plasmids were constructed: S108A, which mutates serine at position 108 of rat EPAC‐1 to alanine; S108E, which mutates serine at position 108 of rat EPAC‐1 to glutamic acid; and an overexpression plasmid without mutations. In vitro, plasmids were transduced into primary neurons after 4–5 days of culture, with 20 μL of HitransG A added to the transduction solution.

### 2.5 MTT Assay

The MTT assay was employed to assess cellular vitality, adhering to the protocol provided by the kit producer. The neuronal cells were subjected to a 4‐h exposure with the MTT reagent solution, followed by the dissolution of the formed formazan precipitates in dimethyl sulfoxide. Subsequent to this, the absorbance was recorded at a wavelength of 570 nm with a Thermo Microplate reader.

### 2.6 Live‐Dead Cell Staining

The variously treated cell samples were subjected to a staining process involving a solution composed of 5 μL calcein AM alongside 20 μL ethidium homodimer‐1, under dark conditions, for a duration of 30 min. Subsequently, the cellular imagery was obtained through a fluorescence microscope (Nikon, Japan).

### 2.7 Western Blot Analysis

Primary neuronal cells and the cortical regions adjacent to the hemorrhagic areas in brain tissue were gathered. Aliquots of the samples, containing 10–50 μg of protein, underwent separation via 10% SDS‐PAGE and were then blotted onto nitrocellulose membranes (Merck Millipore, USA). Membranes were pretreated with a blocking solution of 5% bovine serum albumin for an hour before being exposed to the relevant primary antibodies at a temperature of 4°C throughout the night. On the subsequent day, the membranes were subjected to further incubation with secondary antibodies conjugated to either anti‐rabbit or anti‐mouse IgG for a duration of 1 h. The bands were visualized using an ECL kit. The specifics of the antibodies utilized are documented in Table S1.

### 2.8 Co‐Immunoprecipitation (Co‐IP)

Sample preparation and protein determination were the same as for the western blot. After washing the protein A/G magnetic beads three times with lysis buffer, 250 μg of protein solution and 1 μg of the corresponding IP antibody or normal IgG antibody were added and spun at 4°C overnight. The subsequent day, the protein‐antibody‐bead mixture was washed three times by centrifugation, and then the precipitate was denatured with SDS‐PAGE sample loading buffer and boiled for 5 min.

### 2.9 Nissl Staining

Neuronal death and survival of brain tissue around the hematoma were observed using nissl staining. Brain sections were stained with toluidine blue working solution for 40 min, then dehydrated by an alcohol gradient, transparent with xylene, and finally sealed with neutral balsam. Images of the area around the hematoma in the brain tissue were taken with a microscope (Nikon, Japan).

### 2.10 Fluoro‐Jade B (FJB) Staining

Fluoro‐Jade B staining was employed to evaluate the extent of neuronal damage surrounding the hematoma. The brain slices underwent a preprocessing step where they were immersed in an 80% ethanol mixture with 1% sodium hydroxide for 5 min, and subsequently placed in a 70% ethanol solution for 2 min. Following this, the slices were treated with a 0.06% potassium permanganate solution for 10 min before being dyed with a 0.0004% FJB solution for a 20‐min period. Imaging of the sections was conducted using a fluorescence microscope (Zeiss microscope).

### 2.11 RabGEF Activity Assay

Process the samples according to the recommendations of the kit instructions. Information on the kits is provided in Table S2.

### 2.12 Neurobehavioral Tests

Neurobehavioral changes in rats on days 3, 7, and 14 after ICH were evaluated using the modified Garcia score and the rotarod test, and deficits in spatial cognitive function of rats after ICH were examined using the water maze test.

#### 2.12.1 Modified Garcia Score Test

The modified Garcia scale assessment consists of six indicators of spontaneous activity, body proprioception, response to vibrissae touch, climbing, forelimb outstretching, and limb symmetry. Please see Table S3 for specific scoring criteria.

#### 2.12.2 Rotarod Test

The rotarod test placed different groups of rats on the rotarod apparatus and increased the speed from 5 to 40 rpm over a period of 300 s. The time each rat on the rotarod was recorded for statistical analysis.

#### 2.12.3 Morris Water Maze

Drawing from earlier studies [19], a round‐shaped swimming pool was segmented into four equal sections. The rodents were set free from any of the sections, excluding the one with the hidden platform, and given a 60‐s window to locate it. In each trial, the rats were positioned on the submerged platform for a duration of 15–20 s to aid in the acquisition of environmental clues. On the 26th day following ICH, the submerged platform was taken out, and the rats were permitted to swim for a full 60 s from their original starting point.

### 2.13 Measurement of GSH and MDA Levels

Rats were anesthetized and decapitated, and brain tissue surrounding the hematoma was collected. After weighing, the tissues were homogenized in PBS. Glutathione (GSH) levels and malondialdehyde (MDA) content were measured using a GSH assay kit and an MDA assay kit (Beyotime, China), according to the manufacturer's instructions.

### 2.14 Propidium Iodide (PI) Staining

The cells, which underwent various treatments, were subjected to a 30‐min cohabitation with PI, reaching a terminal concentration of 2 μg/mL, under dim conditions at ambient temperature. Subsequently, they were exposed to Hoechst for an additional 15‐min period. The cells were photographed on a fluorescence microscope, and three random images were captured from each field of view under the fluorescence microscope. The cell number was measured with ImageJ software (NIH), and the percentage of cell death is expressed as PI‐positive cells (%).

### 2.15 Transmission Electron Microscope

The cortical tissue around the hematoma was dissected into 1 mm^3^ piece and fixed in glutaric dialdehyde solution for electron microscopy. Electron microscopy analyses were performed by Standard Testing Group Co. Ltd. (Qingdao, China).

### 2.16 Statistical Analysis

The analysis of all collected data was conducted with GraphPad Prism version 10, employing an unpaired two‐tailed student's *t*‐test for the purpose of comparing the differences between the two groups. For the examination of continuous variables across various groups, either a one‐way or a two‐way analysis of variance (ANOVA) was applied. The data met one or more criteria of the D'Agostino and Pearson normality test, the Shapiro–Wilk normality test, and the KS normality test. The results are presented as the mean ± standard deviation, and a *p*‐value less than 0.05 was deemed to indicate statistical significance.

## 3 Results

### 3.1 Significant Enrichment of the cAMP Pathway Identified in High‐Throughput Sequencing After ICH in Rats

**FIGURE 1:**
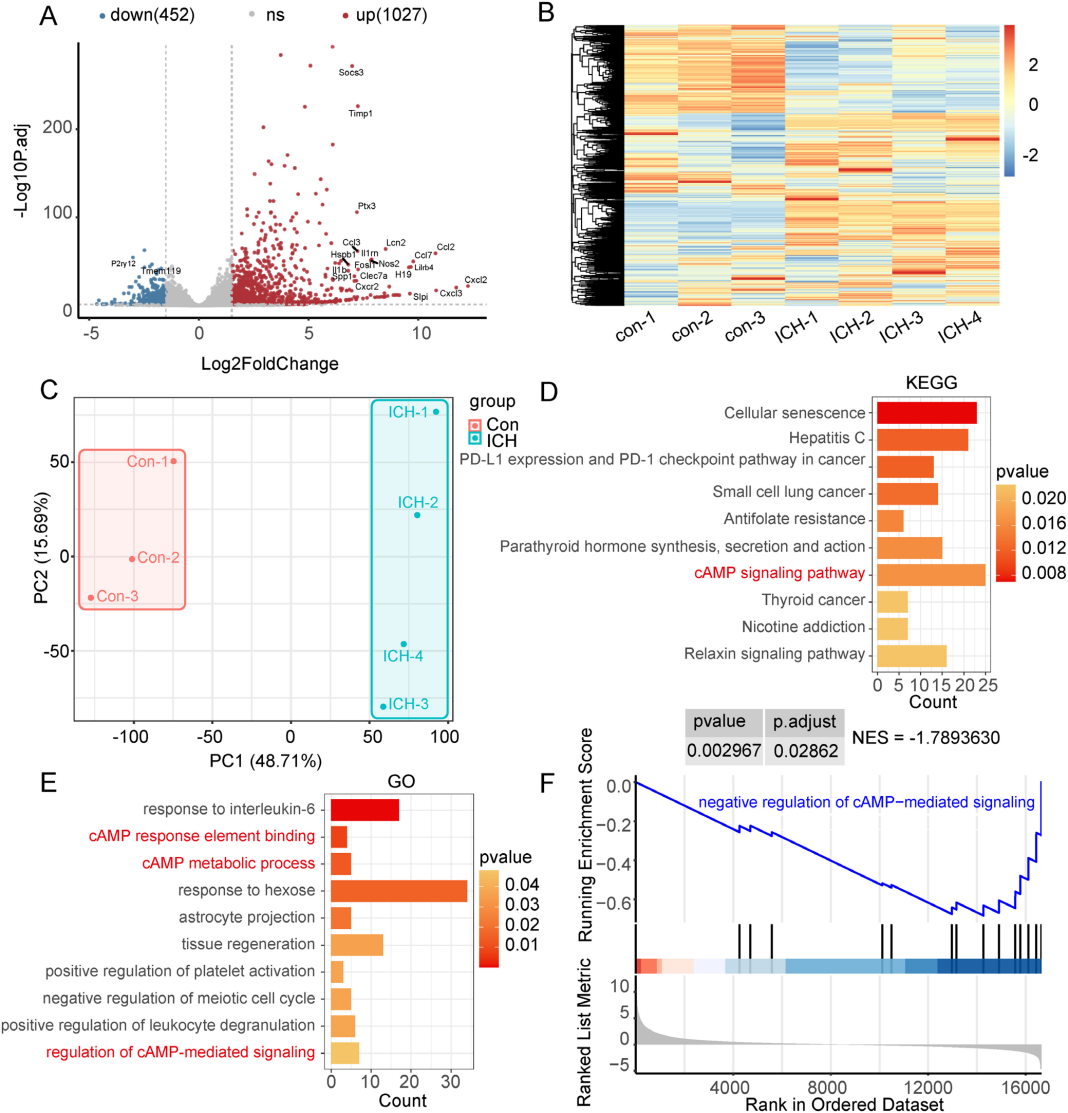
Significant enrichment of the cAMP pathway identified in high‐throughput sequencing after ICH in rats. (A) Volcano plot showing changes in genes. Red dots indicate upregulated genes, blue dots are downregulated genes, and gray dots are genes with no significant modifications. (B) Heat map showing expression of differentially expressed genes between samples. (C) Principal component analysis. (D) KEGG pathway enrichment analysis of differentially expressed genes, shown as bar graphs. (E) GO enrichment analysis of differentially expressed genes, represented by bar chart graphs. (F) Gene set enrichment analysis revealed changes in the associated functional pathways.

The GSE264394 dataset from the GEO database was screened for analysis. The volcano plot illustrates the distribution of differentially expressed genes after ICH, with 20 randomly selected genes shown (Figure 1A). Heatmaps display the clustering of differentially expressed genes in the Sham group and the ICH group (Figure 1B) while principal component analysis further confirmed that the ICH group was significantly different from the Sham group (Figure 1C). Gene Ontology (GO) and Kyoto Encyclopedia of Genes and Genomes (KEGG) analyses revealed that the cAMP pathway and other related pathways may play an important role in the regulation of cellular functions after ICH (Figure 1D,E). Gene Set Enrichment Analysis further indicated significant up‐regulation of the cAMP pathway after ICH (Figure 1F). Overall, these findings suggest that the cAMP signaling pathway may have important biological significance following ICH.

### 3.2 ICH‐Induced EPAC‐1 Activity Was Elevated, While EPAC‐1 Phosphorylation Levels Were Reduced

**FIGURE 2:**
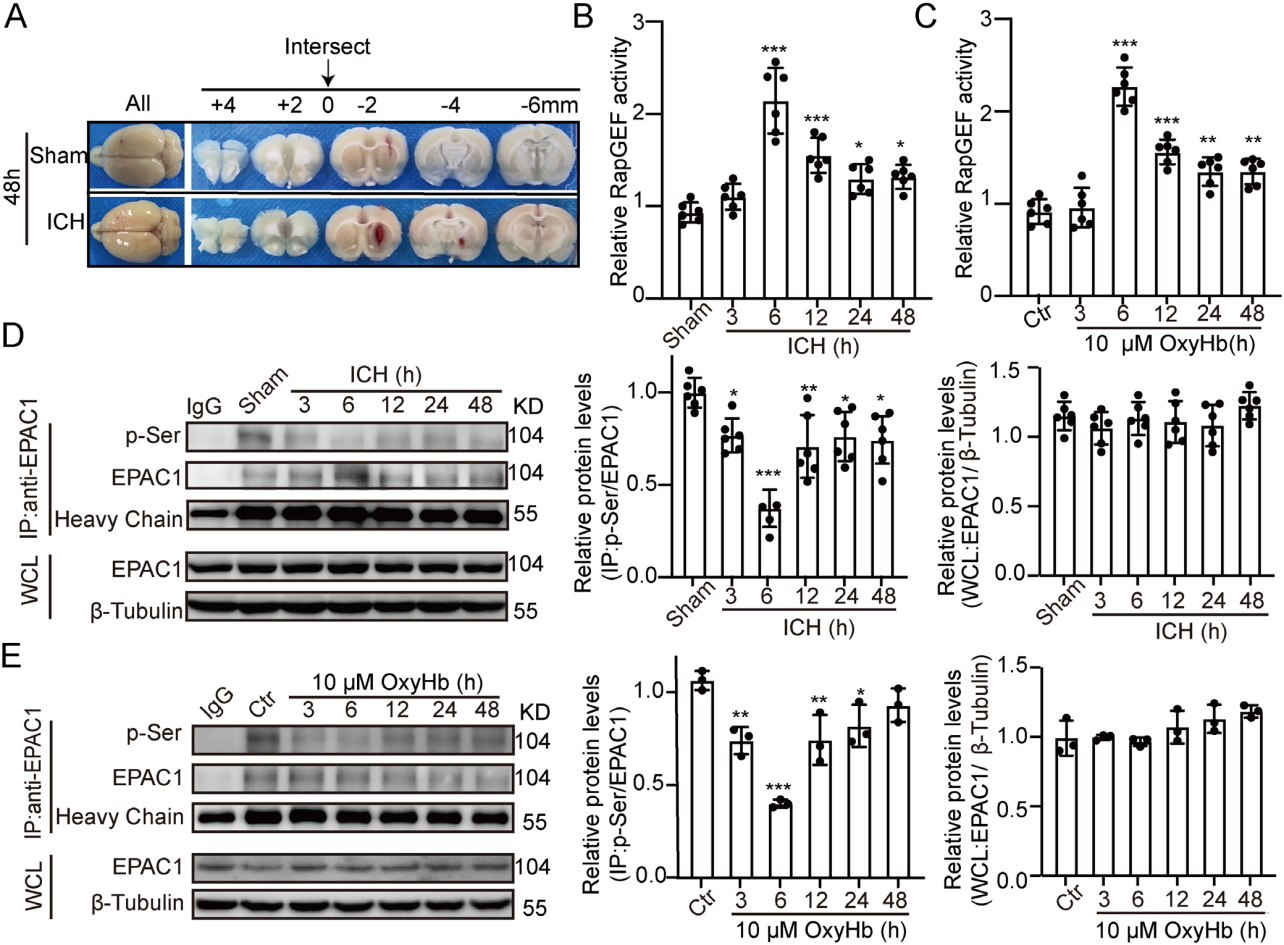
ICH‐induced EPAC‐1 activity was elevated, while EPAC‐1 phosphorylation levels were reduced. (A) Coronal brain sections of rats in the Sham groups and ICH groups. (B) Changes in RabGEF activity at different time points after ICH. (C) Changes in RabGEF activity at different time points after 6 h of OxyHb stimulation. (D) Western blot and quantitative analysis of changes in EPAC‐1 serine phosphorylation at different time points after ICH, *n* = 6. (E) Western blot and quantitative analysis of changes in EPAC‐1 serine phosphorylation at different time points after 6 h of OxyHb stimulation, *n* = 6. All data are presented as mean ± standard deviation. Statistical significance was determined using one‐way analysis of variance (ANOVA) with Tukey's multiple comparisons test, *p < 0.05, **p < 0.01, ***p < 0.001 vs. Sham/Control group.

An ICH model was established on SD rats, with brain images of the Sham group and ICH group shown in Figure 2A. cAMP influences a range of cellular processes by activating EPAC and subsequently Rap1 [20]. RapGEFs are key regulators of Rap GTPases, with EPAC being one of them [21]. Based on previous studies indicating that EPAC‐1 protein levels remain unchanged after ICH [22], we focused on examining changes in RabGEF activity at various time points in vivo and in vitro. Notably, EPAC‐1 activity significantly increased after ICH, peaking at 6 h (Figure 2B,C). It has been reported that G protein‐coupled receptor kinase 2 inhibits EPAC‐1 to Rap1 signaling by phosphorylating the Ser‐108 site in the EPAC‐1 domain [23]. To further investigate whether EPAC‐1 is phosphorylated after ICH, we analyzed that phosphorylation levels significantly decreased at various time points post‐ICH, reaching their lowest point at 6 h compared to the Sham group (Figure 2D). This reduction in EPAC‐1 Ser phosphorylation was also confirmed through in vitro immunoprecipitation experiments (Figure 2E). In summary, our findings indicate that EPAC‐1 activity is markedly upregulated, along with a significant reduction in Ser phosphorylation after ICH.

### 3.3 Activation of EPAC‐1 Phosphorylation Inhibits OxyHb‐Induced Neuronal Death in Primary Cultured Neurons

**FIGURE 3:**
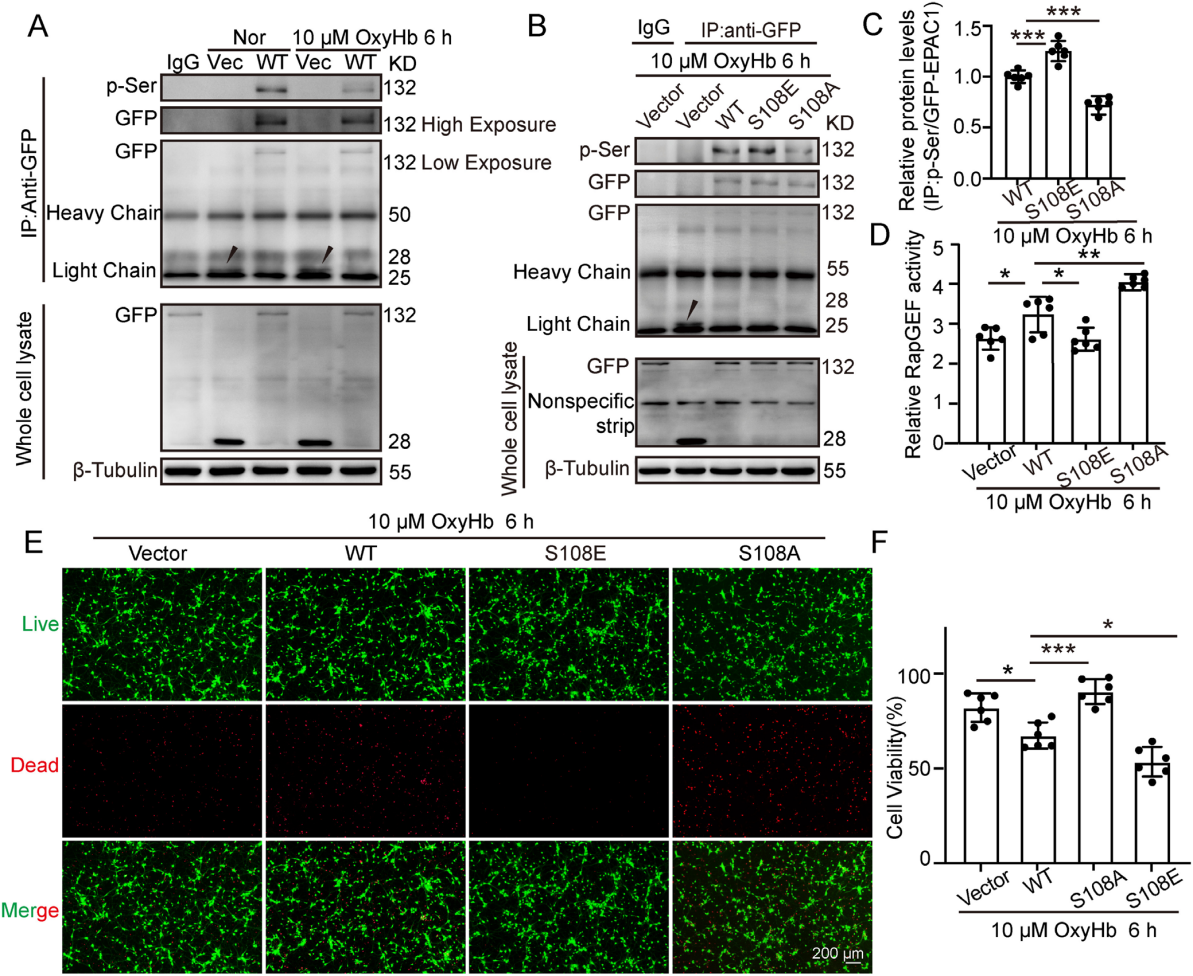
Activation of EPAC‐1 phosphorylation inhibits OxyHb‐induced neuronal death in primary cultured neurons. (A) Changes in phosphorylation of exogenous GFP‐EPAC‐1 after ICH. (B, C) After 6 h of OxyHb stimulation, the plasmid effect and transfection of EPAC‐1 phosphorylation activation and inactivation mutations were verified and quantified. (D) Changes in RabGEF activity in different intervention groups after 6 h of OxyHb stimulation. (E) Live‐dead cell staining was conducted to assess neuronal cell death. (F) Cell viability was assessed using the MTT assay, *n* = 6. All data are presented as mean ± standard deviation. Statistical significance was determined using one‐way analysis of variance (ANOVA) with Tukey's multiple comparisons test, *p < 0.05, **p < 0.01, ***p < 0.001.

To evaluate the effect of EPAC‐1 Ser‐108 phosphorylation on ICH, we constructed plasmids for EPAC‐1 overexpression (WT), a phosphorylation‐activating mutation (S108E) and a phosphorylation‐inactivating mutation (S108A). To verify whether the level of exogenous GFP‐EPAC‐1 phosphorylation would occur and alter after ICH, it was found that the phosphorylation level of EPAC‐1 was significantly reduced after OxyHb stimulation compared with the Vector group, which was aligned with the endogenous changes (Figure 3A). The transfection effects of EPAC‐1 phosphorylation activation and inactivation mutations are shown in Figure 3B. It was found that the phosphorylation level of GFP‐EPAC1 in the S108E point mutation was significantly elevated, while the phosphorylation level of GFP‐EPAC1 in the S108A point mutation was significantly reduced compared to the WT group (Figure 3C). Correspondingly, the activity of neuronal RapGEF was affected by changes in EPAC‐1 phosphorylation, with phosphorylation activation leading to a decrease in RapGEF activity (Figure 3D). Additionally, live‐dead cell staining and cell viability assays demonstrated enhanced cell viability and reduced neuronal death in the S108E group compared to the WT group, while the opposite effect was observed in the S108A group (Figure 3E,F). The results indicate that an increased phosphorylation level of EPAC‐1 Ser‐108 could reduce OxyHb‐induced neuronal cell death.

### 3.4 EPAC‐1 Phosphorylation‐Activated Mutations Reverse ICH‐Induced Neuronal Death in Rats

**FIGURE 4:**
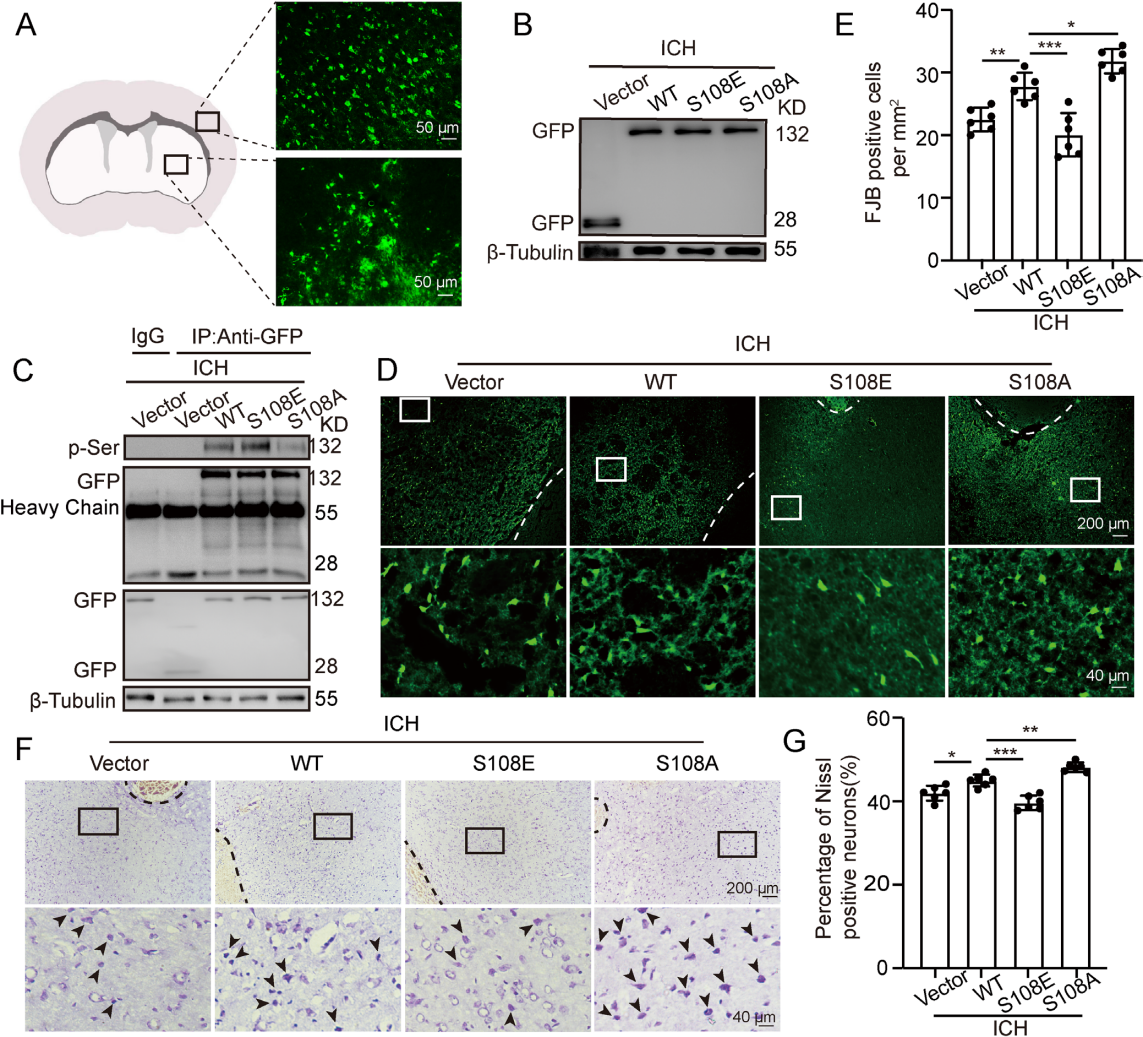
EPAC‐1 phosphorylation‐activated mutations reverse ICH‐induced neuronal death in rats. (A, B) Validation of the effectiveness of in vivo viral interventions. (C) Plasmid effects and transfection of EPAC‐1 phosphorylation activation and inactivation mutations were verified after ICH. (D, E) FJB staining indicated the presence of FJB‐positive cells and quantified the number of cells that were FJB‐positive in the area surrounding the hematoma, *n* = 6. The white dotted line shows the area of the hematoma. The white rectangular box is the enlarged portion of the image below. (F, G) Nissl staining was used to assess cortical neuron morphology and to quantify abnormal nissl bodies in cortical areas, *n* = 6. The black dotted line shows the area of the hematoma. Black arrows indicate Nissl‐positive cells, neuron‐dead cells. The black rectangular box is the enlarged portion of the image below. All data are presented as mean ± standard deviation. Statistical significance was determined using one‐way analysis of variance (ANOVA) with Tukey's multiple comparisons test, *p < 0.05, **p < 0.01, ***p < 0.001.

We utilized lentiviral packaging plasmids for in vivo infection to induce overexpression and site‐specific mutations of EPAC‐1. The effect of viral transfection is shown in Figure 4A,B. Co‐IP assay detected changes in the phosphorylation level of EPAC‐1 at the Ser‐108 position in brain tissues. Compared with the ICH + WT group, phosphorylation activity was significantly enhanced in the ICH + S108E group, while it was significantly reduced in the ICH + S108A group (Figure 4C). To further investigate the role of EPAC‐1 phosphorylation in ICH, we assessed the effects of EPAC‐1 phosphorylation on neuronal death using Nissl and FJB staining (Figure 4D–F). The results indicated that activation of EPAC‐1 Ser‐108 phosphorylation resulted in a significant reduction in both Nissl‐positive and FJB‐positive cells compared to the ICH + WT group, whereas inactivation of EPAC‐1 Ser‐108 phosphorylation aggravated neuronal cell death (Figure 4E–G). In conclusion, activation of EPAC‐1 Ser‐108 phosphorylation could reduce ICH‐induced neuronal death in rats.

### 3.5 The EPAC‐1 Phosphorylation‐Activated Mutation Enhances Neurological Recovery in Rats Following ICH

**FIGURE 5:**
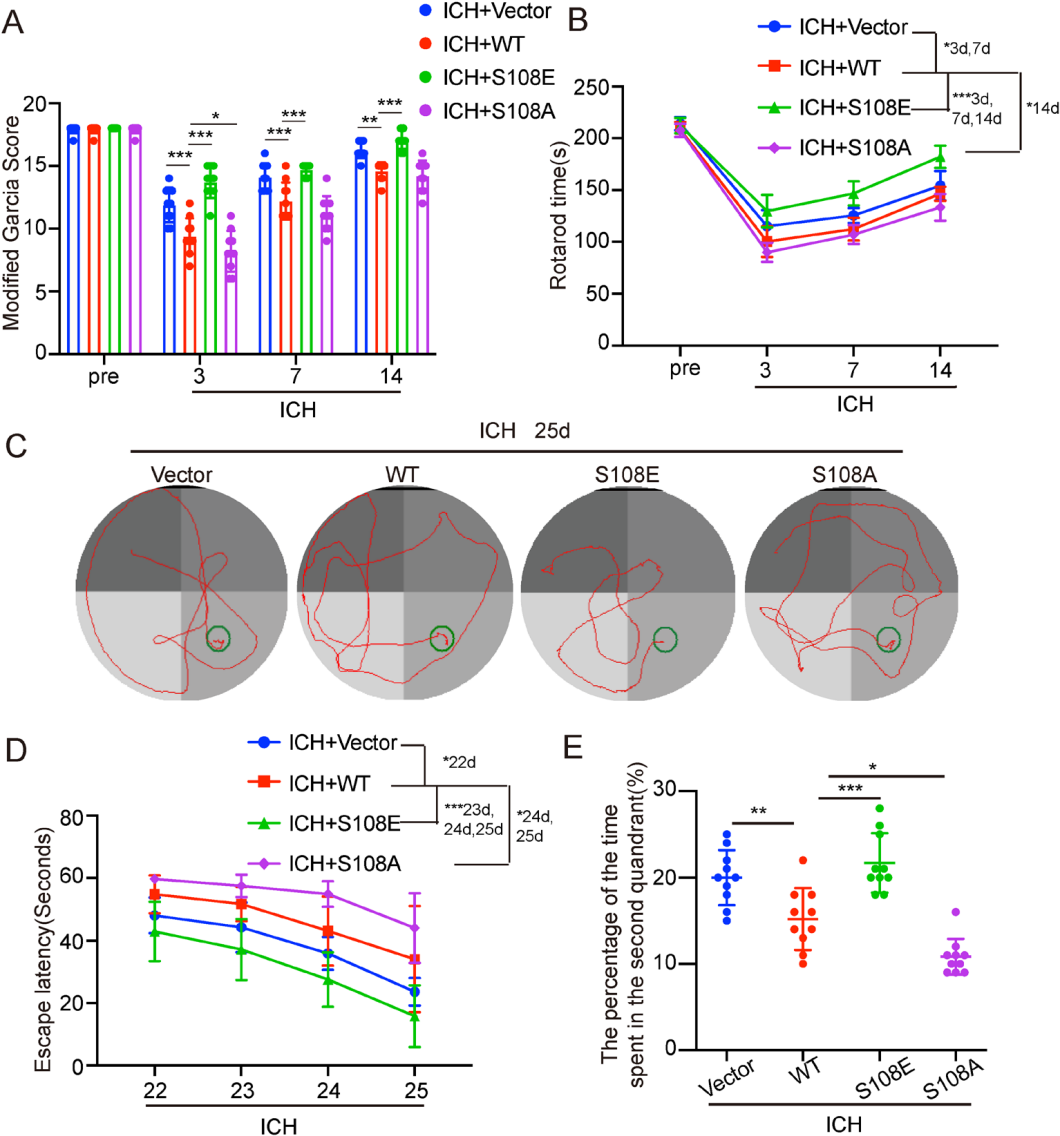
The EPAC‐1 phosphorylation‐activated mutation enhances neurological recovery in rats following ICH. (A) The modified Garcia Score test was used to assess the neurobehavioral impairment in rats. (B) The rotarod test. (C) Exemplary swimming paths of rats in the 25th d water maze test. (D) Time to reach the platform in the water maze test 22–25 days after ICH in each group. (E) Percentage of time rats passed through the quadrant where the platform was located within 1 min after removing the platform on 26 day. All data are presented as mean ± standard deviation. Statistical significance was determined using two‐way analysis of variance (ANOVA) with Tukey's multiple comparisons test, *p < 0.05, **p < 0.01, ***p < 0.001, *n* = 10.

The modified Garcia score and rotarod test were employed to gauge the neurological improvement in rats at the 3‐, 7‐, and 14‐day marks post‐ICH, whereas the Morris water maze was utilized to measure the spatial recognition impairments. Both the modified Garcia score and the rotarod test showed that rats in the ICH + S108E group significantly improved their neurobehavioral function and spent more time on the rotarod (Figure 5A,B). In the Morris water maze, the ICH + WT group exhibited severe behavioral dysfunction compared to the ICH + Vector group (Figure 5C). Rats in the ICH + S108E group required less time and distance to find the platform and took longer to traverse the quadrant after exiting the platform on day 26, while the opposite results were observed in the ICH + S108A group (Figure 5D,E). Therefore, activation of EPAC‐1 Ser‐108 phosphorylation after ICH can effectively improve neurological function recovery in rats.

### 3.6 EPAC‐1 Phosphorylation Activates Mutations That Inhibit Ferroptosis in Neurons by ICH

**FIGURE 6:**
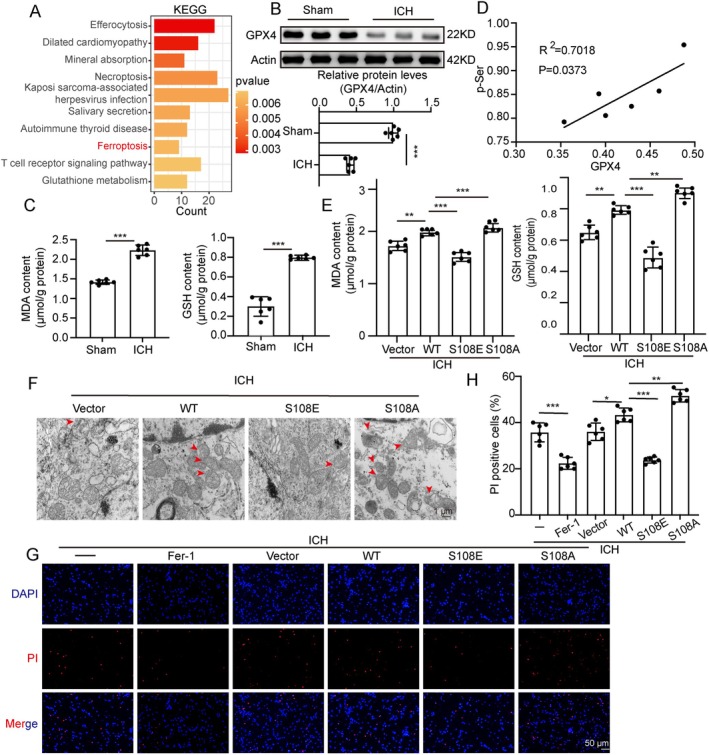
EPAC‐1 phosphorylation activates mutations that inhibit ferroptosis in neurons by ICH. (A) KEGG pathway enriched for ferroptosis, shown as a bar graph. (B) Western blot and quantitative analysis of GPX4 expression after ICH, *n* = 6. (C) Relative content of MDA and GSH between Sham group and ICH group, *n* = 6. (D) Spearman correlation between p‐Ser and GPX4 expression, *n* = 6. (E) Relative content of MDA and GSH among groups, *n* = 6. (F) Mitochondrial structures were examined by means of transmission electron microscopy with a scale bar of 1 μm. Red arrows indicate damaged mitochondria. (G) PI staining between different groups. (H) Quantitative analysis of PI‐positive cells. All data are presented as mean ± standard deviation. Statistical significance was determined using one‐way analysis of variance (ANOVA) with Tukey's multiple comparisons test, *p < 0.05, **p < 0.01, ***p < 0.001.

To delve into the role of EPAC‐1 phosphorylation in the process of neuroprotection, we conducted an additional enrichment analysis on the mentioned dataset, aiming to uncover the involvement of the ferroptosis pathway. Ferroptosis is defined as a controlled type of cell demise, marked by the buildup of harmful iron‐related lipid peroxides to a fatal extent. Following ICH, KEGG enrichment analysis highlighted the ferroptosis pathway's significance (Figure 6A). By measuring the levels of GPX4, MDA, and GSH, we confirmed the occurrence of ferroptosis after ICH (Figure 6B,C). In addition, we performed a correlation analysis between GPX4 and EPAC‐1 Ser phosphorylation after ICH, which showed a statistically significant relationship (Figure 6D). MDA and GSH levels were significantly lower in the ICH + S108E group compared to the ICH + WT group (Figure 6E). Electron microscopy results revealed that, compared to the ICH + Vector group, the mitochondria in the ICH + WT group exhibited more severe damage, characterized by mitochondrial shrinkage and cristae fragmentation. The ICH + S108A group showed even more pronounced mitochondrial damage than the WT group. In contrast, mitochondrial morphology in the ICH + S108E group was significantly improved compared to the WT group, with less shrinkage and more intact cristae, as indicated by arrows (Figure 6F). PI staining indicated that the ICH + S108E group exhibited significantly reduced neuronal cell death compared to the ICH + WT group, with effects comparable to those observed with the addition of Fer‐1 (ferroptosis inhibitor) (Figure 6G,H).

## 4 Discussion

Within the scope of this research, our initial exploration into the function and underlying process of EPAC‐1 in relation to neuronal damage was conducted utilizing rat autologous ICH and primary neuronal OxyHb simulation models. Under our findings: (1) EPAC‐1‐Rap1 was activated in neuronal cells and the level of phosphorylation at the EPAC‐1 Ser‐108 was reduced and lowest at 6 h after ICH. (2) Phosphorylation of EPAC‐1 at Ser‐108 plays a neuroprotective role, attenuating neuronal cell death and ameliorating neurological deficits post‐ICH, while suppression of this phosphorylation exacerbates neuronal damage. (3) Activation of EPAC‐1 phosphorylation at Ser‐108 also inhibits ferroptosis in neurons. Collectively, these results highlight the critical role of EPAC‐1 Ser‐108 phosphorylation in promoting neuronal survival following ICH.

As a ubiquitous second messenger, cAMP is a pivotal signaling molecule within cells, and its downstream effector molecules, including protein kinase A (PKA) and EPAC, mediate distinct functions across various tissues [24, 25]. The research advantages of EPAC over PKA in the context of ICH are notable for several reasons: First, EPAC is abundantly expressed in immune cells and modulates signaling pathways distinct from those regulated by PKA. This grants EPAC a unique and significant role in modulating inflammatory responses, maintaining blood–brain barrier integrity, and promoting neuronal repair following ICH [26]. Second, EPAC plays a role in regulating intracellular calcium homeostasis and oxidative stress responses during the calcium dysregulation and oxidative injury induced by ICH, potentially offering neuroprotection through these mechanisms [27]. Finally, the specific functions of EPAC within the nervous system remain incompletely understood, providing a valuable opportunity for researchers to uncover new signaling pathways and therapeutic targets.

EPAC‐1 functions as a specific guanine nucleotide exchange factor (GEF) for Rap1. It activates Rap1 by facilitating the exchange of GDP (inactive) for GTP, allowing Rap1 to act as a key signaling molecule involved in processes such as cell survival, proliferation, and differentiation [21, 28]. Previous studies have demonstrated that EPAC‐1 undergoes phosphorylation at Ser‐108, which inhibits its ability to activate Rap1 [23]. In brief, phosphorylation of EPAC‐1 Ser‐108 can function as an “on/off switch” to control the proper activation of Rap1‐GTP, which is essential for the maintenance of normal neuronal function.

In our research, we demonstrated through both in vivo and in vitro experiments that the phosphorylation level of EPAC‐1 at Ser‐108 decreased following ICH. Activation of EPAC‐1 phosphorylation at Ser‐108 has been shown to inhibit neuronal cell death and significantly improve neurological outcomes after ICH by modulating this phosphorylation. To further investigate the underlying mechanisms, we found that the modulation of EPAC‐1 phosphorylation may be linked to ferroptosis. Our findings confirmed that activation of EPAC‐1 phosphorylation at Ser‐108 effectively inhibited ferroptosis in neurons.

Neuronal ferroptosis is a key mechanism of cell death following ICH, characterized by intracellular iron accumulation, lipid peroxidation, and disruption of antioxidant defense mechanisms [29]. Although direct studies on the relationship between EPAC‐1 and ferroptosis are still limited, evidence suggests that EPAC‐1 may influence ferroptosis by regulating intracellular antioxidant defenses and iron homeostasis [30]. EPAC‐1 can mitigate oxidative stress after ICH by activating antioxidant defense mechanisms [31]. Since the accumulation of free iron promotes ferroptosis post‐ICH, EPAC‐1 may help regulate iron homeostasis by influencing the expression of hepcidin or iron transport proteins, thus controlling intracellular iron levels [32]. In summary, EPAC‐1 Ser‐108 phosphorylation exerts neuroprotective effects after ICH, partly by reducing ferroptosis. This mechanism likely involves a synergistic effect of reducing oxidative stress and modulating iron metabolism pathways.

Nevertheless, the present research possesses several constraints that warrant attention in subsequent scholarly endeavors. For instance, factors such as the sex and age of the experimental rats should be considered, as well as the potential influence of other blood components when simulating ICH in vivo. Additionally, the underlying mechanisms of EPAC‐1 phosphorylation and its role in neuronal ferroptosis should be further investigated. In brief, EPAC‐1 phosphorylation may serve as a novel mechanism underlying neuronal injury after ICH, and screening small‐molecule compounds targeting EPAC‐1 phosphorylation could provide promising candidates for clinical ICH treatment.

## 5 Conclusion

The phosphorylation of EPAC‐1 is crucial for the maintenance of neuronal survival, potentially associated with the inhibition of ferroptosis following ICH. These findings provide potential therapeutic targets for the clinical treatment of ICH.

## Author Contributions

Xiang Li, and Wanchun You conceived the study and designed experiments. Guannan Jiang and Yan Zhuang performed experiments and data analysis. Guannan Jiang wrote the manuscript. Jialei Zhou helped analyze bulk RNA sequencing data. Gang Chen and Siyuan Yang edited and revised the manuscript. All authors provided feedback on the manuscript and approved the final paper.

## Consent

All authors have read and approved the manuscript.

## Conflicts of Interest

The authors declare no conflicts of interest.

## Supporting information


**Table S1.** Antibodies.
**Table S2.** Reagents.
**Table S3.** The modified Garcia score criteria for the sub‐tests.

## Data Availability

The datasets used and/or analyzed during the present study are available from the corresponding author upon reasonable request.
