# Supplementary material for: Phosphorylation of cAMP‐Activated Exchange Protein‐1 Participates in Neuroprotection and Ferroptosis Regulation Following Intracerebral Hemorrhage in Rats
Source: CNS Neurosci Ther. 2025 Apr 9;31(4):e70373. doi: 10.1111/cns.70373 (PMC11979711; doi:10.1111/cns.70373)
Supplement: Supplementary file 1 — Table S1. Antibodies. Table S2. Reagents. Table S3. The modified Garcia score criteria for the sub‐tests. [file CNS-31-e70373-s001.docx]

| **Antibody** | **Full name** | **Specificity** | **Citation** | **Factory** | **Host** | **Dilution** |
| --- | --- | --- | --- | --- | --- | --- |
| Epac1 | Anti-Epac1 Antibody | Mouse, Rat, Human | ab109415 | Abcam | Rabbit | 1:2000 |
| Epac1 | Anti-Epac1 Antibody | Human | ab21236 | Abcan | Rabbit | 1:2000 |
| EPAC1 | Anti-Epac1 Antibody | Mouse, Rat, Human | 4155s | CST | Mouse | 1:2000 |
| Rabbit IgG | Anti-rabbit IgG Antibody | Mouse, Rat, Human | 7074s | CST | Rabbit | 1:2000 |
| Mouse IgG | Anti-mouse IgG Antibody | Mouse, Rat, Human | 7076s | CST | Mouse | 1:2000 |
| Tubulin | Tubulin beta Antibody | Mouse, Rat, Human | AF7011 | YESEN | Rabbit | 1:2000 |
| GFP | Anti-GFP/eGFP-tag Antibody | Mouse, Rat, Human | AP52804 | Abcepta | Mouse | 1:1000 |
| EPAC1 | Anti-Epac1 Antibody | Mouse, Rat, Human | sc-28366 | Santa Cruz | Mouse | 200 µg/ml |
| PSPH | PSPH Rabbit Antibody | Mouse, Rat, Human | 14513-1-AP | Proteintech | Rabbit | 400 µg/ml |
| Normal mouse IgG | Normal mouse IgG | ----- | sc-2025 | Santa Cruz | Mouse | 200µg/0.5ml |
| Actin | Beta actin | Human,Mouse,Rat | 30102ES60 | YESEN | Rabbit | 1:2000 |
| GPX4 | Anti-Glutathione Peroxidase 4 Antibody | Mouse, Rat, Human | ab125066 | Abcam | Rabbit | 1:2000 |

**Appendix Table 1 Antibodies**

**Appendix Table 2** **Reagents**

| **Reagents** | **Full name** | **Density** | **Citation** | **Factory** |
| --- | --- | --- | --- | --- |
| Protein A/G | Protein A/G Magnetic Beads for IP | 10 mg/mL | B23202 | Selleck |
| LIVE/DEAD® | LIVE/DEAD® Viability/Cytotoxicity Kit | 5nM | 2481050 | Invitrogen |
| Lipo 3000 | Lipofectamine™ 3000 | ---- | L3000001 | Invitrogen |
| GEF Exchange Assay Kit | GEF Exchange Assay Kit | 30/T | BK100 | Cytoskeleton |
| MTT | MTT Cell Proliferation and Cytotoxicity Assay Kit | 5mg/ml | C0009S | Beyotime |
| FJB | Fluoro-Jade®B Dry Powder | 30mg | TR-150-FJB | Biosensis |
| Nissl | Toluidine Blue | **-----** | 89640-5G | Sigma-aldrich |

**Appendix Table 3 The modified Garcia score criteria for the sub-tests**

| **Sub-test** | **0** | **1** | **2** | **3** |
| --- | --- | --- | --- | --- |
| **Spontaneous Activity (SA)** | No movement | Barely moves | Moves but does not approach at least three sides of cage | Moves and approach≥3 walls of cage |
| **Body Proprioception (BP)** | No response | Unilateral response | A weak bilateral response | A brisk bilateral response |
| **Response to vibrissae touch (VT)** | No response | Unilateral response | A weak bilateral response | A brisk bilateral response |
| **Climbing (CL)** | No movement | Failed to climb or circled | Climbed to the top with a weak grip | Climbed to the top with a strong grip |
| **Forelimb Outstretching (FO)** | A  paretic  forelimb | Walked in circles | Walked asymmetrically or to one side | Walked symmetrically on forepaws |
| **Limb Symmetry (LS)** | Hemiparesis | Left forelimb or left hindlimb flexed | Asymmetric extension | Extended symmetrically |
